# Comparing different models to forecast the number of mass shootings in the United States: An application of forecasting rare event time series data

**DOI:** 10.1371/journal.pone.0287427

**Published:** 2023-06-26

**Authors:** Xue Lei, Cameron A. MacKenzie

**Affiliations:** Department of Industrial and Manufacturing Systems Engineering, Iowa State University, Ames, IA, United States of America; PLoS ONE, UNITED STATES

## Abstract

The number of mass shootings in the United States has increased in the recent decades. Understanding the future risk of the mass shootings is critical for designing strategies to mitigate the risk of mass shootings, and part of understanding the future risk is to forecast the frequency or number of mass shootings in the future. Despite the increasing trend in mass shootings, they thankfully remain rare events with fewer than 10 mass shootings occurring in a single year. Limited historical data with substantial annual variability poses challenges to accurately forecasting rare events such as the number of mass shootings in the United States. Different forecasting models can be deployed to tackle this challenge. This article compares three forecasting models, a change-point model, a time series model, and a hybrid of a time series model with an artificial neural network model. Each model is applied to forecast the frequency of mass shootings. Comparing among results from these models reveals advantages and disadvantages of each model when forecasting rare events such as mass shootings. The hybrid ARIMA-ANN model can be tuned to follow variation in the data, but the pattern of the variation may not continue into the future. The mean of the change-point model and the ARIMA model exhibit much more less annual variation and are not influenced as much by the inclusion of a single data point. The insights generated from the comparison are beneficial for selecting the best model and accurately estimating the risk of mass shootings in the United States.

## 1 Introduction

Public mass shootings, in which 4 or more individuals are killed from a shooting in a public setting, are a major social problem in the United States and generate a significant amount of media attention and debate over the best strategies to reduce their risk. The United States accounts for approximately one-third of all public mass shootings in the world [1]. Research in mass shootings finds that the rate of mass shootings in the United States has increased in the 21st century [2–4] although these conclusions depend in part on the definition of mass shootings [5]. Equally as disturbing, the lethality of mass shootings has increased in the 21st century [6, 7]. Much of the existing literature on modeling and forecasting the trend in mass shootings focuses on associating different factors such as poverty, gun laws, gun ownership, and population with the prevalence of mass shootings [4, 8–11]. Historical data on mass shootings have significant variability and with relatively few data points. This poses challenges to accurately forecast the number of mass shootings. Among the existing work, little research has analyzed and compared among different models that can potentially forecast the number of mass shootings in the United States. Therefore, developing sophisticated models that can capture stochastic characteristics of rare events with limited data is needed.

Being able to accurately model and forecast the number of mass shootings in the United States should help us understand and analyze the risk of these events and should lead to more informed discussions of how best to mitigate the risk. Mass shootings are rare events, and accurately forecasting rare events is problematic and statistically challenging [12]. According to the Violence Project [13], the maximum number of mass shootings that has occurred in a single year is 8 with most years since 2000 seeing 2-6 mass shootings. Models that explicitly incorporate uncertainty may be the best approach to forecasting rare events [14, 15]. Bayesian models incorporate uncertainty in both model parameters and future forecasting results [16, 17]. Poisson models can also be appropriate for modeling rare events because the rare events can be considered a recurrent process [18], and the Poisson model does not require normally distributed errors [19]. Attempting to model rare events can also lead to overfitting due to a limited set of data for training the model. Potential solutions to overfitting are penalized regression (e.g., Ridge regression, Lasso regression) [20, 21] and bootstrapping [22, 23].

Several models could be used to forecast the frequency of mass shootings in the United States, but the rare-event nature and annual variability in the number of mass shootings create obstacles to generating an accurate forecast and determining which model is most appropriate. This article compares three models to forecast the annual number of mass shootings, a Bayesian change-point model, the autoregressive integrated moving average (ARIMA) model, and a hybrid of an ARIMA and neural network model. The change-point models time series data through a non-homogeneous Poisson process view. The ARIMA model is a classic time series model which is commonly used for time series modeling. The Hybrid model combines the deep learning model’s advantage into time series modeling. All these three models model time series based on different stand points and use different types of data. Three models were chosen The change-point model fits time series data to a non-homogeneous Poisson process, and mass shootings largely seem to be independent events over time that obey the assumptions of a Poisson process. The ARIMA model is chosen because it is a classic time series model used very frequently to model annual data over several years in which the data exhibits autocorrelation. The hybrid model is a relatively new model that combines deep learning with ARIMA to. All these three models model time series based on different stand points and use different types of data. We compare the fitting and forecasting performance of these models. The comparison helps us learn the advantages and disadvantages of each model to forecast the number of mass shootings. Comparing among the results reveals more general insights into the usefulness of each model for forecasting rare events.

A change-point model detects times when the stochastic process or time series changes. The change-point model often models recurrent events in which the rate of occurrence changes with time [24–28]. Probabilistic methods of change-point models typically follow a Bayesian approach [29, 30] and have been used to measure ozone levels in Mexico City [28], tuberculosis in New York City [24], the risk of teenage drivers [31–34], and the trend in mass shootings [35].

ARIMA is one of the most widely used forecasting models for time series [36–39]. The ARIMA model can express different time series through its flexible parameters [40] and can tackle non-stationary time series [41]. ARIMA models have been applied to predict crime in many countries, including the Philippines [42], Australia [43], China [44], and the United Kingdom [45]. A bivariate ARIMA model is used to investigate the relationship between crime and arrests in Oklahoma City [46], and an ARIMA model studies the impact of COVID-19 stay-at-home orders on the gun violence in Buffalo, New York [47].

ARIMA models may not be ideal for forecasting rare events in part because the ARIMA equation is a linear equation, but some examples exist in the literature of using ARIMA to forecast rare events. An empirically based smoothing technique combined with ARIMA is used to forecast the occurrence of rare events (strong earthquakes in Parkfield, California) [48]. The ARIMA is applied to forecast drought in the Jordan River basin where 0-2 severe droughts occur and 4 moderate droughts occur [49] apply. An resampling strategy is proposed to forecast rare events with an ARIMA mdoel when the training data is imbalanced, which can be a feature of rare events [50]. An autoregressive model combined with a change-point detection model is used to detect outliers in a time series [51].

The third type of model used in this paper to forecast mass shootings is a hybrid of ARIMA and an artificial neural network (ANN). ANN is a popular machine learning tool because of its ability to model nonlinearity [52, 53] and learn from data [54, 55]. Neural networks have been applied to forecast time series of rare events [56–58]. The hybrid ARIMA-ANN model is proposed for time series forecasting [59]. The hybrid ARIMA-ANN model frequently has a better prediction accuracy than either the pure ARIMA model or ANN model [60–63]. Some of the literature finds that the hybrid model performs better than the ARIMA model for time series forecasting based on limited historical data [59, 60, 64]. The ARIMA model considers the linear combinations of inputs for modeling a time series. However, the nonlinear combinations of inputs may also be needed for the time series data. The ANN model is a widely used model to capture nonlinearities in data [65]. The unique advantage of using the hybrid AIMRA-ANN is to model the time series data via a linear part and a nonlinear part.

This article fits the time series of mass shootings in the United States as recorded by the Violence Project [13] from 1966-2020 to each of the three models: a change-point model with a time-dependent rate function, the ARIMA model, and the ARIMA-ANN hybrid model. Such a comparison requires several unique approaches. Since comparing among statistical models often separates data into training and testing sets, the comparison among these models separates the historical data on mass shootings into different training and testing sets while preserving the time series of the data. The hybrid model is relatively new, and we compare its ability to fit historical data and forecast the future with these other models for rare events. The results of this comparison lead to a discussion of the advantages and disadvantages of using each type of model to forecast the annual number of mass shootings. This discussion may be broadly applicable to other types of applications. Comparing these models contributes significantly to our understanding of the risk of mass shootings and forecasting rare events.

Section 2 introduces each of the forecasting models with an explicit focus on the hybrid ARIMA-ANN model because it is less well known. Section 3 compares among the different models, and we examine how choosing a different number of nodes in the hybrid model substantially impacts the performance of this model. We also study the effect of including the number of mass shootings in the most recent year 2020 on the forecast of each model. We conclude in Section 4 with some insights from this study.

## 2 Forecasting models

This section introduces the three models that are used to forecast mass shootings: the Bayesian change-point model, the ARIMA model, and the hybrid ARIMA-ANN model.

### 2.1 Change-point model

A non-homogenous Poisson process (NHPP) refers to a Poisson process where the arrival rate changes over time. A change-point model can use a time-dependent rate function to model a NHPP. Commonly used time-dependent rate functions are the power law process, the Musa-Okumoto process, the Goel–Okumoto process, the generalized Goel-Okumoto process, and the Weibull-geometric process (WG) [66–70]. The change-point model identifies one or more points in time when the parameters of the rate function changes. Bayesian methods can be used to detect change points or more accurately the posterior distribution for these change points [30]. After the change-point model is fit to the historical data, we can generate a probabilistic forecast of future events by simulating the NHPP by sampling parameters from the posterior distribution.

Since the Violence Project data for mass shootings contain the date of each mass shooting, the change-point model with the time-dependent rate function can be fit to the historical data on mass shootings by modeling the time between each mass shooting. Since mass shootings have become more frequent over time, a NHPP is a reasonable model for this event. Lei et al. [35] fit the different time-dependent rate functions to the mass shootings for zero, one, and two change points. They find that the WG rate function performs the best according to three performance metrics: deviance information criterion, marginal likelihood, and residual sum of squares. Thus, we use the change-point model with the WG rate function in this article to model and forecast the annual number of mass shootings. The WG rate function is:

$$\lambda (t)=\frac{\frac{\alpha }{\beta }{\left(\frac{t}{\beta }\right)}^{\alpha -1}}{1-\rho e^{-{\left(\frac{t}{\beta }\right)}^{\alpha }}}$$ (1)

where λ(*t*) is the rate at time *t*, and *α* > 0, *β* > 0, and *ρ* ∈ (0, 1) are parameters of the rate functions.

As explained in [35], this rate function is used to derive the likelihood function for the observed mass shootings data. We assume uniform prior distributions for the parameters in the rate function and the change points. The software package Stan which is run via the R library rstan applies a Markov Chain Monte Carlo sampling technique to generate a posterior distribution for the rate function parameters and the change points.

### 2.2 ARIMA model

The ARIMA model assumes that future observations are linearly dependent on past observations and random errors. The parameters of the non-seasonal ARIMA model are *p*, *d*, and *q*. The parameter *p* is the order of autoregression. The parameter *d* is the differencing number. The parameter *q* is the order of the moving average model [40, 71]. The ARIMA model can be expressed as:

$$\phi \left(B\right)\nabla^{d}\left(y_{t}-\mu \right)=\theta \left(B\right)\varepsilon_{t}$$ (2)

where *y*_*t*_ is the observation at time *t*, *ε*_*t*_ is the random error at time *t*, *μ* is the mean value, and *B* is backward shift operator. The backward shift operator causes the observation that it multiplies to be shifted backwards in time by one period. In our case, *By*_*t*_ = *y*_*t*−1_. The functions $\phi \left(B\right)=1-\sum_{i=1}^{p}\varphi_{i}B^{i}$, $\theta \left(B\right)=1-\sum_{j=1}^{q}\theta_{j}B^{j}$, and ∇^*d*^ = (1 − *B*)^*d*^. The parameters *ϕ*_1_ … *ϕ*_*p*_ are the autoregressive parameters to be estimated. The parameters *θ*_1_…*θ*_*q*_ are the moving average parameters to be estimated. The random errors *ε*_*t*_ are independently and identically distributed with zero mean and a constant variance.

The first step of fitting the ARIMA model to data is choosing the values for *p*, *d*, and *q*. We use the Akaike Information Criterion (AIC) to select the best order of the ARIMA model. An approximate calculation of the AIC is based on the sum of squared residuals (RSS) [61]:

$$\text{AIC}=k[\text{log}(\frac{2\pi \text{RSS}}{n})+1]+2\left(p+q\right)$$ (3)

where *k* is the number of observations in the ARIMA model. Given the order of the ARIMA model, the parameters of model can be estimated by the maximum likelihood estimation [72, 73].

Python software packages pmdarima.arima use auto.arima function to estimate parameters in the ARIMA model [74]. After setting the maximum values for *p* and *q*, the auto_arima function will test all different value combinations of *p* and *q* and select the best one with the smallest AIC. The auto_arima package uses the Augmented Dickey-Fuller test to determine if the time series is stationary [75]. If the time series is not stationary, auto_arima will provide a suitable value of *d*.

### 2.3 Hybrid ARIMA-ANN model

The ARIMA model considers the linear combinations of inputs for modeling a time series. However, the nonlinear combinations of inputs may also be needed for the time series data. The ANN model is a widely used model to capture nonlinearities in data [65]. The unique advantage of using the ANN is there are no prior assumptions about the form of the model. The form of the ANN model depends on the data. The hybrid AIMRA-ANN models the time series data via a linear part and a nonlinear part. The model can be expressed as:

$$y_{t}=L_{t}+N_{t}$$ (4)

where *L*_*t*_ is the linear model and *N*_*t*_ is the nonlinear model at time *t*. The linear model *L*_*t*_ is estimated by the ARIMA model and denoted as $\hat{L_{t}}$.

The residual at time *t*, *e*_*t*_, is obtained by:

$$e_{t}=y_{t}-\hat{L_{t}}$$ (5)

The analysis of residuals indicates whether the ARIMA model fully captures the time series. The nonlinear component of the residuals can be modeled by using the ANN model. The function *h* is generated by the ANN model as a function of the preceding *n* residuals before time *t*:

$$e_{t}=h\left(e_{t-1},e_{t-2},\ldots ,e_{t-n}\right)+\varepsilon_{t}$$ (6)

where *e*_*t*_ is the current residual, *e*_*t*−1_, *e*_*t*−2_, …, *e*_*t*−*n*_ are the *n* most recent residuals before time *t*. The model $\hat{N_{t}}=h\left(e_{t-1},e_{t-2},\ldots ,e_{t-n}\right)$ is the estimate of *e*_*t*_. Residuals should be normalized and mapped to the range [0, 1] before being input in the ANN model.

**Fig 1:**
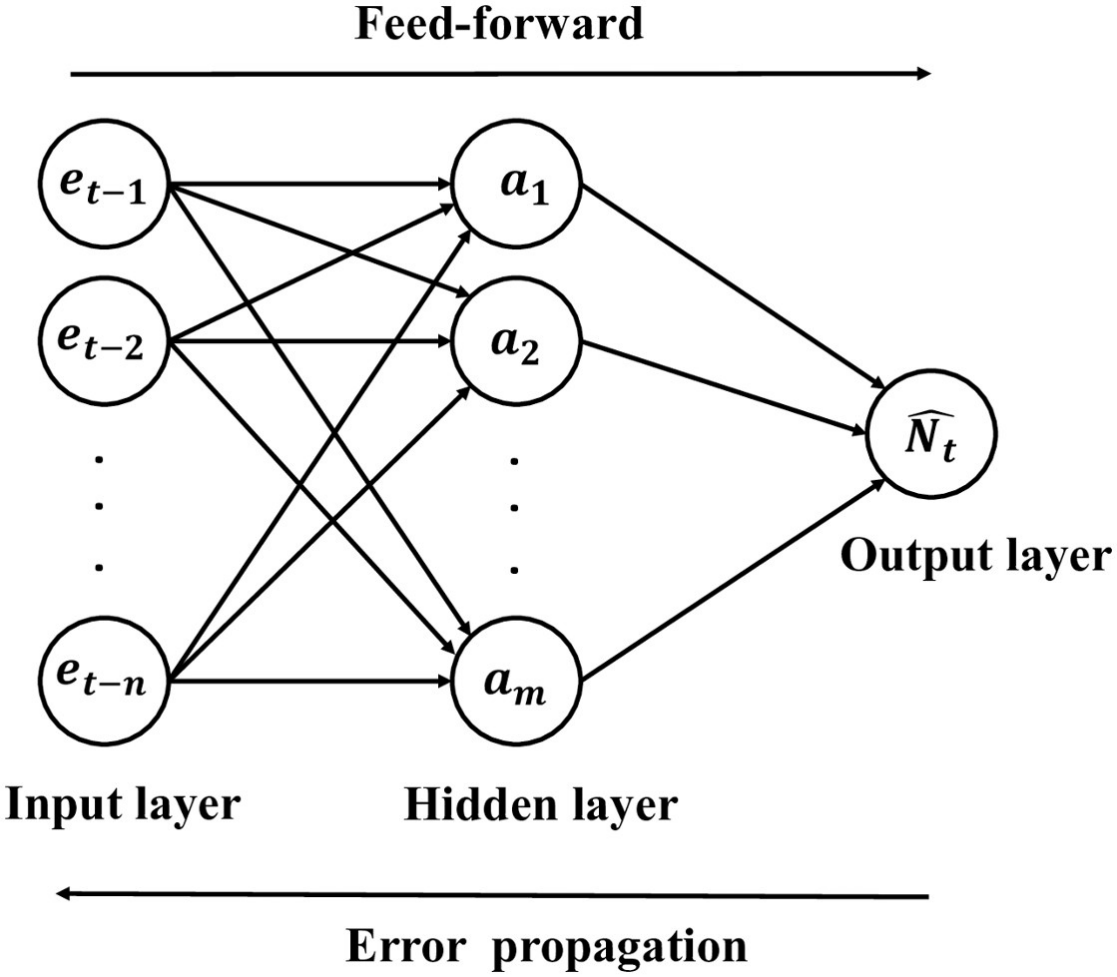
Single hidden layer neural network.

The architecture of the ANN model is very flexible [76]. Three types of layers exist in the ANN model. The input layer consists of different inputs. The output layer exports the outputs of the model. The hidden layer connects the input layer and output layer. Unlike the input and the output layer, the hidden layer can have more than one layer. The most commonly applied ANN structure is the single hidden layer and back propagation ANN [77]. In this research, the ANN model estimates the current residual *e*_*t*_ based on the previous *t* − 1 residuals. The input layer has multiple input nodes. The output layer only has one output node. Multiple hidden nodes exist in the hidden layer. The general ANN architecture considered in this paper is shown in Fig 1.

The activation functions embedded in the ANN model allow the model to capture nonlinearity. The activation functions used for each node define the output of that node through some inputs. Many different activation functions can be used in the ANN model, such as the sigmoid (Sig) function, the hyperbolic tangent (Tanh) function, the SoftPlus function, and the binary step function [78–81]. The Sig and Tanh functions are used as the activation functions for the hidden layer and the output layer, respectively, in this article. The form of these two activation functions are:

$$\begin{array}{c} \text{Sig}\left(x\right)=\frac{1}{1+\text{exp}(-x)} \end{array}$$ (7)

$$\begin{array}{c} \text{Tanh}\left(x\right)=\frac{1-\text{exp}(-2x)}{1+\text{exp}(-2x)} \end{array}$$ (8)

The mathematical relations between the three layers in the Fig 1 can be described by the activation functions. There are *I* data points to train the ANN model for the nonlinear part of the annual count of mass shootings. For data point *i* (*i* ∈ *I*), ${\text{x}}^{\left(i\right)}=\left(e_{t-1}^{\left(i\right)},e_{t-2}^{\left(i\right)},\ldots ,e_{t-n}^{\left(i\right)}\right)$ is the output of the input layer where *n* is the number of nodes in the the input layer, or more simply, the number of inputs. The corresponding output of the hidden layer is ${\text{a}}^{\left(i\right)}=\left(a_{1}^{\left(i\right)},a_{2}^{\left(i\right)},\ldots ,a_{m}^{\left(i\right)}\right)$, where *m* is the number of nodes in the hidden layer. The relationship between the input layer and the hidden layer is:

$${\text{a}}^{\left(\text{i}\right)}=\text{Tanh}({\text{W}}^{\left[1\right]}{\text{x}}^{\left(\text{i}\right)}+b^{\left[1\right]})$$ (9)

where **W**^[**1**]^ and *b*^[1]^ are the parameters for the hidden layer. Similarly, the relationship between the hidden layer and the output layer is:

$${\hat{N_{t}}}^{\left(i\right)}=\text{Sig}({\text{W}}^{\left[2\right]}{\text{a}}^{\left(\text{i}\right)}+b^{\left[2\right]})$$ (10)

where **W**^[**2**]^ and *b*^[2]^ are the parameters for the output layer. The cost function used for back propagation to update all parameters should be a measurement of accuracy, such as the mean squared error *J* [82]:

$$J=\frac{1}{I}\sum_{i=1}^{I}(e_{t}^{\left(i\right)}-{\hat{N_{t}}}^{\left(i\right)}{)}^{2}$$ (11)

where $e_{t}^{\left(i\right)}$ is true residual at time time *t* for data point *i* as obtained from the ARIMA model.

A potential problem raised with the ANN model is overfitting. Overfitting often happens when the model has a complex structure and many parameters. Regulation methods can reduce the effect of the problem. The regulation term can be added to the cost function to prevent forming a large neural network. The regulation term penalizes large weights and results in fitting a less complex model. Another way to avoid overfitting is to reduce some nodes of the hidden layer [83]. This dropout method frequently performs better than adding a regulation term for complex neural networks, but adding a regulation term is easier to apply. Since the ANN model in this research only has a single hidden layer, it is not too complex. An *L*2 regulation term is added to the cost function:

$$\begin{array}{cc} J_{regularized} & =J+L2\\ & =\frac{1}{I}\sum_{i=1}^{I}(e_{t}^{\left(i\right)}-{\hat{N_{t}}}^{\left(i\right)}{)}^{2}+\frac{\lambda }{2}({\text{W}}^{[1]⊺}{\text{W}}^{\left[1\right]}+{\text{W}}^{[2]⊺}{\text{W}}^{\left[2\right]}) \end{array}$$ (12)

**Fig 2:**
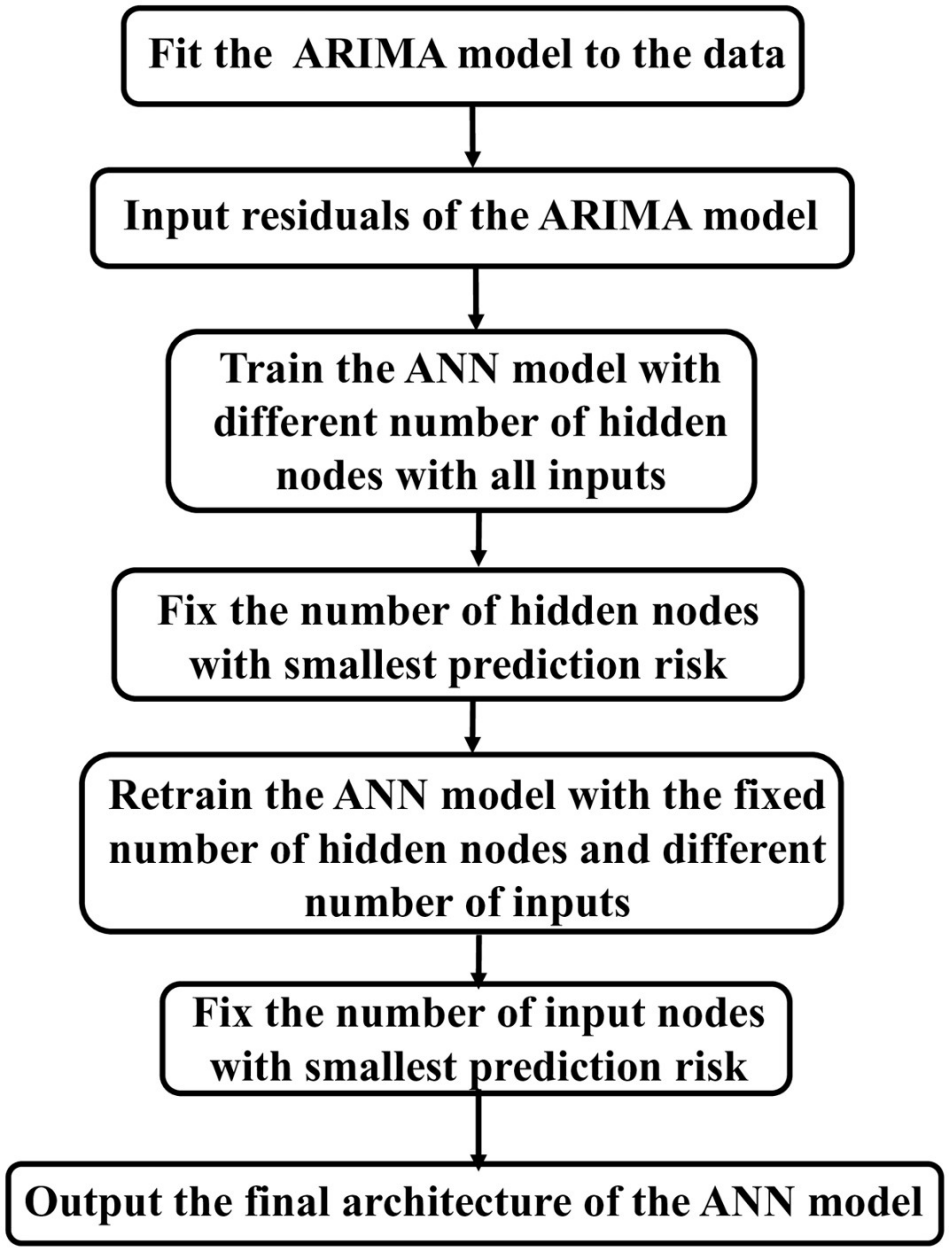
The architecture selection of the ANN model.

Another problem that needs to be solved is selecting the number of input nodes *n* and the number of hidden nodes *m* shown in Fig 1. It is time consuming to try every different combination of *n* and *m*. Different methods have been proposed to find the optimal architecture of the ANN model [84–86]. One architecture selection strategy suggests a sequential network construction (SNC) [87]. The SNC for the ANN model is depicted in Fig 2. This process can be summarized in two steps. The first step is to select the number of hidden nodes, and the second step is to select choosing the number of input nodes given the hidden nodes.

The prediction risk represents the expected prediction performance of the model. By comparing the prediction risk of different models, we can select the model with the best generalization ability. The general definition of prediction risk is the expected mean squared error for the test data set. In many cases, calculating the expected value of the mean squared error is challenging because of a limited test set. Hence, we need to estimate the prediction risk. Other methods to estimate predication risk include cross validation and algebraic estimation [88–91]. We let the ANN model train over all of the data and calculate the prediction risk by the algebraic estimation. The estimation based on all available data is:

$$\hat{P}=J*(1+\frac{2Q}{I})$$ (13)

where $\hat{P}$ is the estimated prediction risk, *J* is the mean squared error of the ANN model trained over all available data, and *Q* is the number of weights used in the ANN model. Based on the single hidden layer neural network shown in Fig 1, *Q* = *n* × *m* + *m*.

## 3 Comparing among different forecasting models

The data of mass shootings are available from different sources. The commonly used mass shootings data sources are New York City Police Department (NYCPD) [92, 93], FBI [94], Mother Jones [95], Gun Violence Archive [96], and Violence Project [13]. One of the model types used to estimate the number of mass shootings in the United States—change-point models—assumes that mass shootings is a non-homogeneous Poisson process (NHPP). These models require the time between each incident in a unit of time as small as possible. The Violence Project databases provide the day of each shooting. The Violence Project also provides a long observation period, from 1966-2019. Given these reasons, this research uses the mass shootings data from the Violence Project [13].

Table 2 in the Appendix shows the annual count of mass shootings recorded by the Violence Project from 1966 to 2019. The first mass shooting recorded by the Violence Project took place on August 1, 1966, which corresponds to the starting time in the change-point model *t*_1_ = 0. The ARIMA and hybrid ARIMA-ANN models use the annual number of shootings rather than the number of days between each shooting.

The Violence Project data on mass shootings covers the years 1966-2019. In order to compare the forecast accuracy among the three models, it is necessary to divide the data into a training set and a testing set. Since the data is a time series, randomly dividing the data into a training and testing set is incorrect. Instead, the training set is established as the annual number of mass shootings from 1966 to year *T* and the testing set is the annual number of mass shootings from year *T* + 1 to 2019. The final year *T* of the training set varies during this analysis, and the proportion of years in the testing set ranges from 10% to 30% of the total number of years. Our comparison among the three models analyzes the root mean squared error (RMSE) and mean absolute percentage error(MAPE) on the training set and on the testing set data and also explores how the models perform when forecasting the annual number of mass shootings in the future.

### 3.1 Comparison of model performance with different size training sets

The last year of the training set *T* changes from 2003 to 2014. For each training set and its corresponding test set, we fit three different types of forecasting models, the change-point model with the WG rate function, the ARIMA model, and the hybrid ARIMA-ANN model. The Python package pmdarima.arima is used to select *p*, *q*, and *d* for the ARIMA model for each training set. We limit the domain of *p* and *q* to be between 0 and 5 and the domain of *d* to be between 1 and 3. The auto_arima, which is imported into the Python package, selects *p* = 0, *q* = 1, and *d* = 1 for all of the training sets. Given the ranges of these parameters, the ARIMA(0-1-1) model results in the best fit for the data.

For the hybrid model, ARIMA(0-1-1) is used to model the linear part of the hybrid ARIMA-ANN model. The inputs for the ANN model are the residuals from the ARIMA(0-1-1) model. Each training set may provide a different architecture for the ANN model. As shown in Fig 2, the number of hidden nodes is selected before the number of input nodes. The maximum number of nodes in the hidden layer is set to 10 and the ANN model is trained with a different constraint on the maximum number of input nodes, 3, 5, 7, or 10. For each training set, the best architecture of the ANN model is based on the prediction risk calculated by Eq 13. The first step trains the fully connected ANN model with all the available input nodes (*n* = 3, 5, 7, or 10) and varies the number of hidden nodes *m* from 0 to 10. The number of hidden nodes *m* is selected with the smallest prediction risk when the number of input nodes *n* is fixed at 3, 5, 7 or 10. Then we fix the number of hidden nodes *m* at selected value. The ANN model is then retrained with the number of inputs ranging between 0 and the maximum number of input nodes (3, 5, 7 or 10). The number of input nodes *n* is chosen for the ANN model with the smallest prediction risk. This architecture selection process is repeated for each training set with the years of the training ranging from 1966-2003 to 1966-2014. The architecture selection results for different training sets when we consider the different maximum numbers of input nodes presented in the Appendix, Tables 4–7.

**Fig 3:**
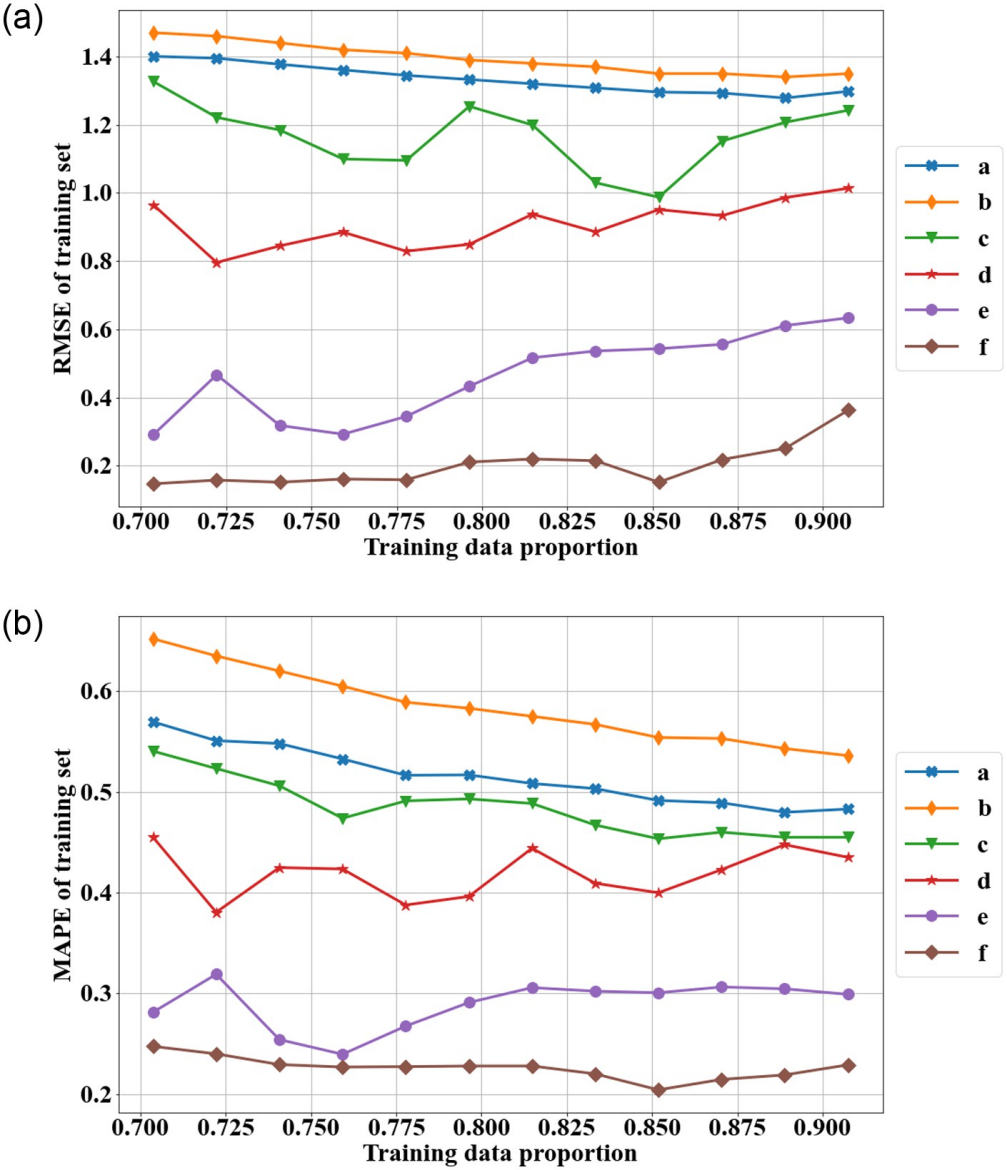
The training RMSE and MAPE of different models over different training sets (a: change-point model with WG rate function, b: ARIMA model, c: hybrid ARIMA-ANN with maximum 3 input nodes, d: hybrid with maximum 5 input nodes, e: hybrid with maximum 7 input nodes, f: hybrid with maximum 10 input nodes).

RMSE and MAPE are used to compare the different models’ performances over the different sizes of the training set [97]. Fig 3a displays the training RMSE for each model with the various size of training set data. The RMSE and MAPE for the change-point model with the WG rate function are based on the mean annual counts of the model. The hybrid ARIMA-ANN model always has the smallest RMSE and MAPE over the different training sets. The change-point model with the WG rate function and the ARIMA model have very similar performance for the training set data, and the RMSE and MAPE decreases for both models as the training set gets larger except for the largest training set (years 1966-2014). The maximum number of input nodes affects the training RMSE and training MAPE for the hybrid ARIMA-ANN model. The ANN model with a largest maximum number of input nodes (10) has the smallest training RMSE and training MAPE.

**Fig 4:**
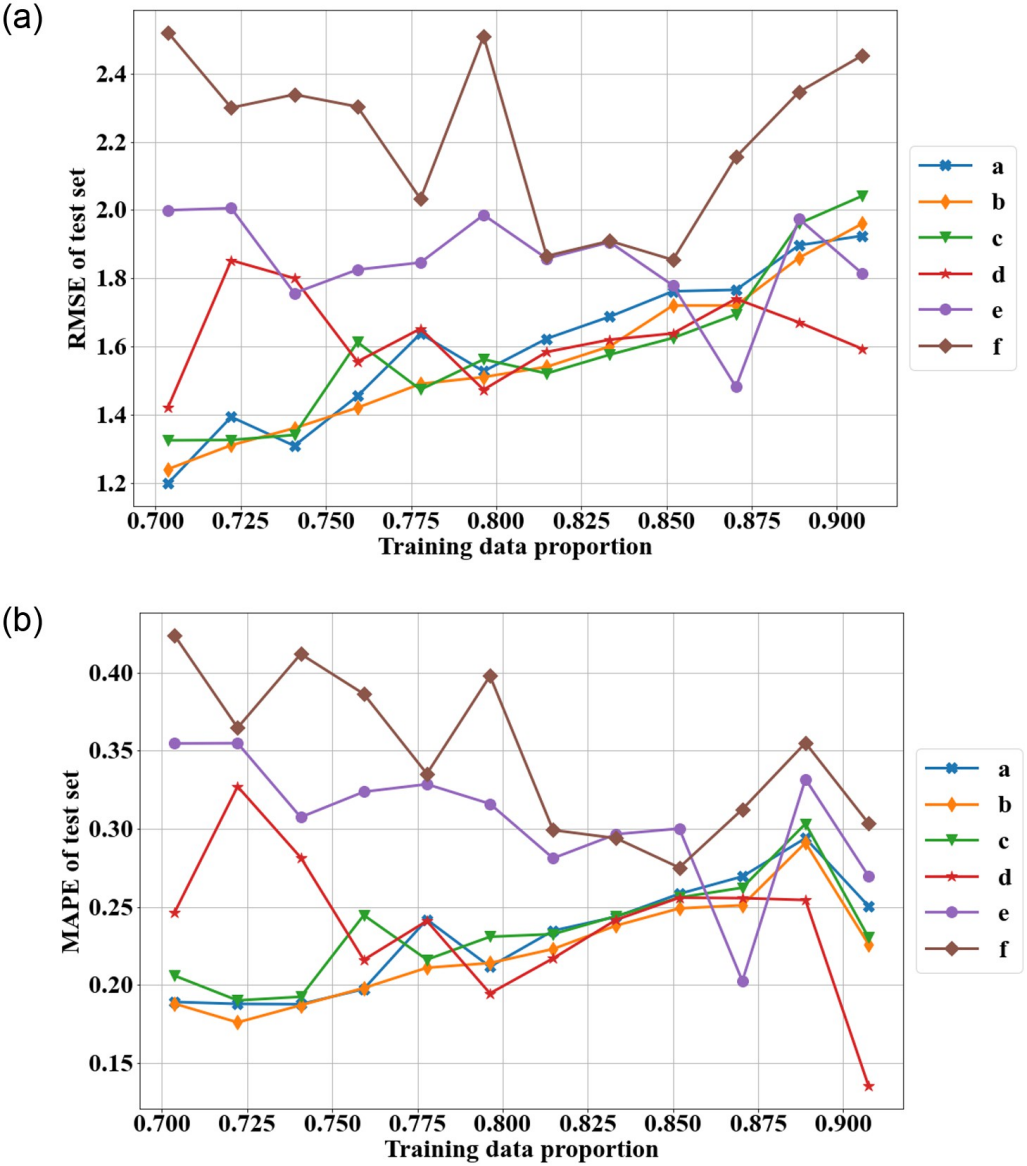
The test RMSE and MAPE of different models over different training sets (a: change-point model with WG rate function, b: ARIMA model, c: hybrid ARIMA-ANN with maximum 3 input nodes, d: hybrid with maximum 5 input nodes, e: hybrid with maximum 7 input nodes, f: hybrid with maximum 10 input nodes).

Fig 4 depicts the test RMSE and MAPE for each model with the different training sets. The test RMSEs and MAPEs for the change-point model, the ARIMA model, and the ARIMA-ANN model with a maximum of three input nodes generally increase as the size of the testing set decreases. The other hybrid ARIMA-ANN models (maximum 5, 7, and 10 input nodes) may have overfitting issues. Although these models have the smallest training RMSE, they frequently have the largest test RMSEs and MAPEs. The hybrid model with a maximum of 5 input nodes looks to perform the best out of all the models when the testing begins with years 2014 or 2015, and the test RMSE and test MAPE remain relatively constant for the different testing sets.

**Fig 5:**
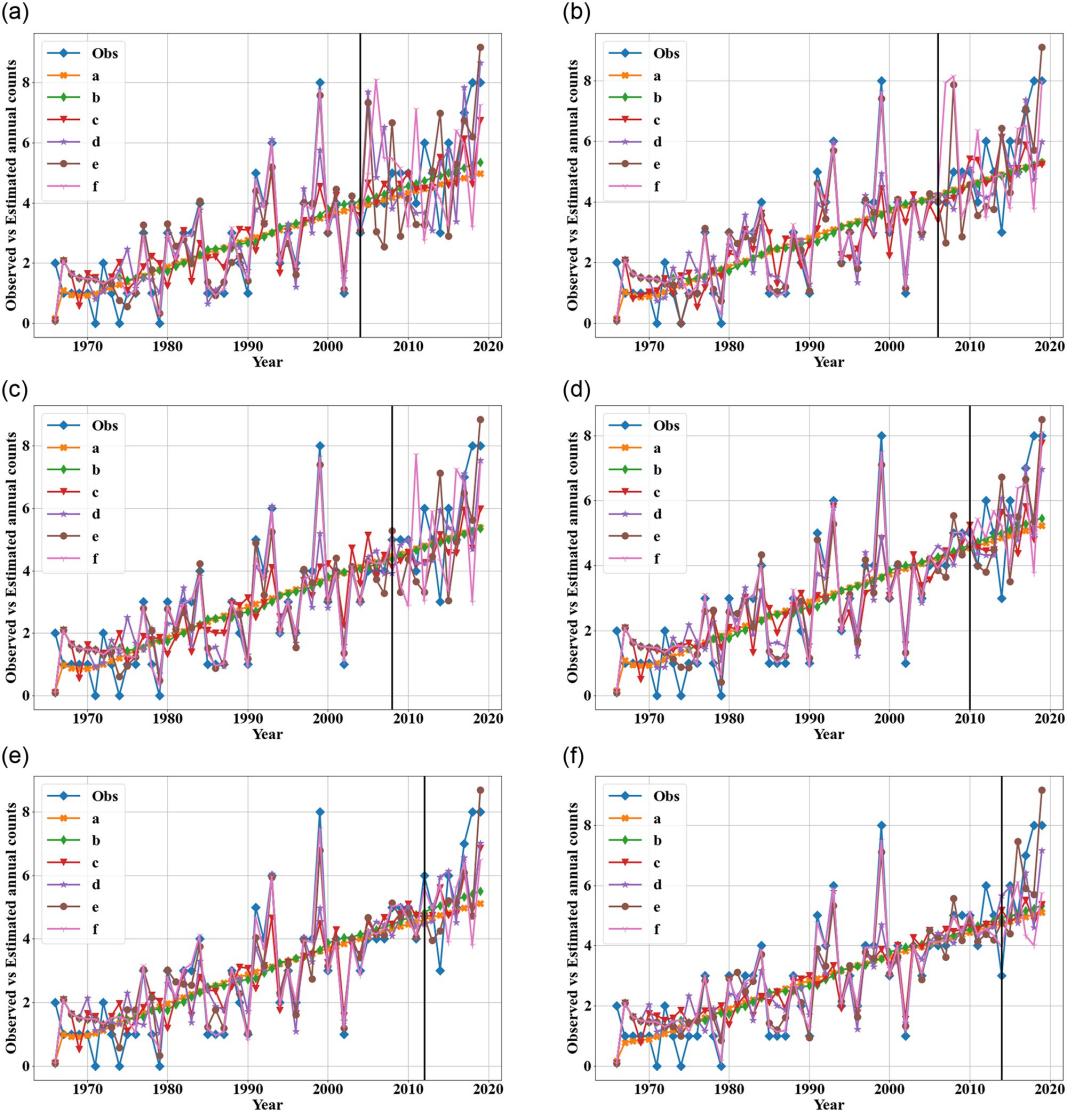
Observed and estimated annual counts from different models with using different training sets (obs: real observations from the Violence Project database, a: change-point model with WG rate function, b: ARIMA model, c: hybrid ARIMA-ANN with maximum 3 input nodes, d: hybrid with maximum 5 input nodes, e: hybrid with maximum 7 input nodes, f: hybrid with maximum 10 input nodes).

The training RMSE, MAPE and test RMSE, MAPE provide exact errors on how the different models fit the mass shootings data from the Violence Project database. Comparing the models’ outputs with the annual number of mass shootings enables us to understand the results more intuitively. Fig 5 depicts some plots showing these comparisons. The plot for the change-point model depicts the mean annual counts from the model. The change-point model and the ARIMA model provide very similar estimates and capture the increasing trend in the number of mass shootings. While the ARIMA model generally suggests almost a linear trend over time with little variation, the hybrid ARIMA-ANN model follows the variation of the annual counts of mass shootings quite well for the training set data. The hybrid model is trying to capture a pattern in the variation from year to year. Although the testing sets also depict substantial annual variation, there is not really a pattern. The hybrid models, especially those models with a greater maximum number of input nodes, correctly forecast substantial variation in the annual number of mass shooting, but they generally fail to forecast accurately if a year will have fewer (i.e., 3 or 4) mass shootings or more (i.e., 7 or 8) mass shootings.

According to the above comparison, the hybrid ARIMA-ANN models with a maximum of 7 and 10 input nodes may suffer from overfitting. The large test errors(RMSE and MAPE) for these two hybrid models indicate that the fluctuation pattern of annual shootings does not continue in the same way. The number of mass shootings in a year exhibits a lot of randomness, which is difficult if not impossible to forecast accurately. The hybrid ARIMA-ANN model with a maximum of 5 input nodes generates good RMSE and MAPE for both the training and testing sets, and perhaps this model appropriately balances between reflecting the trend in mass shootings and capturing some of the variation. Another way to forecast the variation in mass shootings is with a prediction interval for the ARIMA model or a credible interval of the change-point model.

### 3.2 Forecasting results for the future

**Fig 6:**
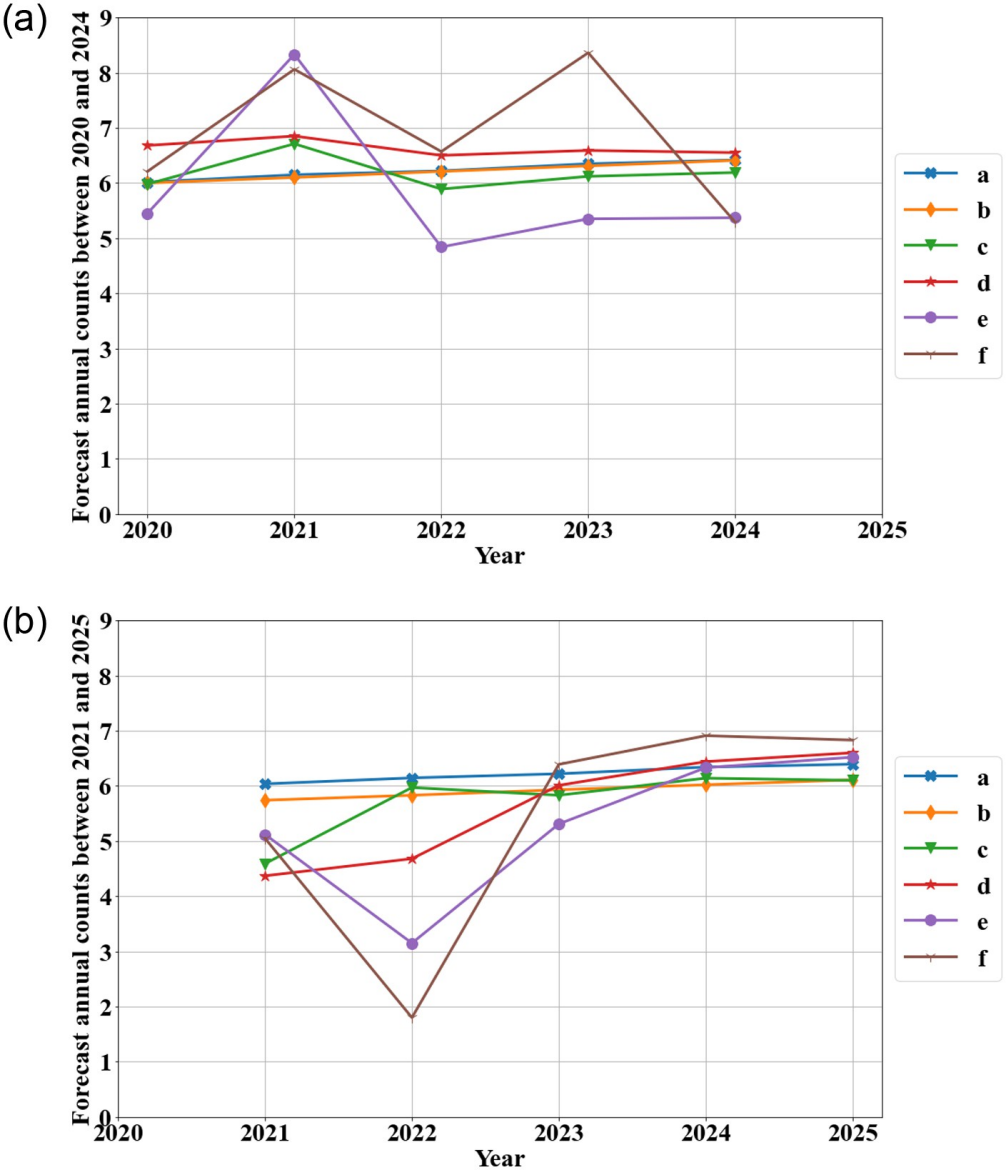
Forecasting of the annual counts of mass shootings (a: change-point model with WG rate function, b: ARIMA model, c: hybrid ARIMA-ANN with maximum 3 input nodes, d: hybrid with maximum 5 input nodes, e: hybrid with maximum 7 input nodes, f: hybrid with maximum 10 input nodes).

In addition to using testing sets comprised of historical data to compare the models results, we also analyze how the models use the entire set of data to forecast the number of mass shootings 5 years into the future. Each model is trained on the data from 1966 to 2019 in order to forecast mass shootings from 2020 to 2024. The Violence Project recently completed its data for mass shooting in 2020, a year in which only one mass shooting occurred. Each model is also trained on the data from 1966 to 2020 in order to forecast mass shootings from 2021 to 2025. Comparing the forecast of 2020-2024 and the forecast of 2021-2025 can provide insight into the sensitivity of the models to a recent change (1 mass shooting in 2020). Fig 6(a) shows the forecasted number of mass shootings in each year from 2020 to 2024 based on the historical data from 1966 to 2019. The change-point model, the ARIMA model, and the hybrid models with a maximum of 3 or 5 input nodes predict a relatively constant number of mass shootings (between 6 and 7 shootings). The hybrid models with a maximum of 7 or 10 input nodes forecast much more variation with approximately 8 mass shootings in 2021 but only 5 in 2024. The two models’ forecasts diverge in 2023 as their forecasts differ by approximately 3 shootings.

As depicted in Fig 6(b), the hybrid models with a maximum of 7 and 10 input nodes are very sensitive to the additional data point of one mass shooting in 2020. These two models have similar forecasts to the other four models in 2021, but the two models forecast a relatively small number of mass shootings (approximately 3 shootings for the 7-input-node model and 2 shootings for the 10-input-node model) in 2022. The other four models predict between 4.5 and 6.5 mass shootings in 2022. All six models forecast a relatively similar number of mass shootings (approximately 6±1 shootings) for the years 2023-2025. Each of the six models that included the data point from 2020 forecasts fewer shootings than the same model if the data point from 2020 is not included. A sudden and recent decrease in the number of mass shootings impacts all of the models’ forecasts although it impacts the hybrid models with a large number of inputs the most. Because the change point model and the ARIMA model capture the overall trend of mass shootings. Since the ANN part of the hybrid model is used to model the residual of the ARIMA model. The Hybrid model is more sensitive to the data variation(recent change in data).

The prediction interval of a forecasting model provides a range in which the future observation will fall with a certain probability. The wider prediction interval means more uncertainty exists in the forecast. We compare the prediction intervals of the forecasted number of mass shootings in 2020 given the data from 1966-2019. We also compare the prediction intervals for 2021 when the data of 2020 is included in training set. Table 1 depicts the 95% prediction intervals estimated by different models in 2020 and 2021.

**Table 1:** Prediction intervals in 2020 and 2021.

Model	2020	2021
Change-point model(WG)	[2, 12]	[2, 12]
ARIMA	[3.33, 8.66]	[2.80, 8.68]
ARIMA-ANN (max 3 input nodes)	[3.74, 8.22]	[1.98, 7.20]
ARIMA-ANN (max 5 input nodes)	[4.21, 9.15]	[1.66, 7.08]
ARIMA-ANN (max 7 input nodes)	[1.71, 9.19]	[1.96, 8.28]
ARIMA-ANN (max 10 input nodes)	[2.96, 9.46]	[1.73, 8.37]

The ARIMA-ANN model with 3 input nodes provides the narrowest prediction interval for the forecasts. The width of prediction interval for the change-point model is the widest, which is likely due to the highly skewed posterior distribution in the change-point model. Including the single mass shooting in 2020 changes the models’ prediction intervals except for that of the change-point model. The change in 2020 brings more uncertainty with the forecasts of the ARIMA model and the ARIMA-ANN models with 3 or 5 input nodes. The change in 2020 decreases the widths of the prediction intervals for the ARIMA-ANN models with 7 or 10 input nodes. Including another data point in these relatively wide prediction intervals decreases the uncertainty in these two models’ forecasts.

## 4 Conclusion

This paper compares the performance of different models to forecast the annual number of mass shootings. Three types of models are compared, the change-point model with a WG rate function, the time series ARIMA model, and the hyrbid ARIMA-ANN model. The hybrid model has four different variants, depending on the maximum number of input nodes. The last year of the training set is varied in order to analyze the performance of the models on slightly different testing sets while keeping the time series elements of the data intact. The models’ forecasts for the first half of the decade of the 2020s are compared especially as it relates to whether or not the number of mass shooting in 2020 is included.

The main limitation of this article is the comparison among these models to a single data set, the historical data on mass shootings. Applying these types of forecasting models to multiple time series, especially time series data on other rare events, would enable us to make stronger conclusions about the benefits and drawbacks of each modeling approach. Other time series data with similar rates of frequencies could be severe natural disasters in the United States, armed military conflicts, and fatal aviation accidents. Another potential limitation is that several factors may contribute to the frequency of mass shootings such as population, gun legislation, and the prior occurrence of mass shootings. Although including some of these factors may improve the forecast of mass shootings, such a modeling approach would also require the ability to forecast the prevalence of those factors into the future.

Since this paper only examines the performance of these models on one data set, making sweeping conclusions about when each type of model should be used may not be wise. However, the performance and forecasting results can provide more general insights into the advantages and disadvantages of these models and specific insights into the annual number of mass shootings. The hybrid ARIMA-ANN model, especially if the ANN model has a large number of input nodes, fits the training set time series the best. The hybrid model reflects the substantial variation in the historical data of annual mass shootings. Conversely, the ARIMA model depicts a relatively stable trend over time and its RMSE for the training set is the largest of all of the models. The mean of the change-point model depicts a very consistent trend over time. As a probabilistic model, the change-point model’s distribution also reflects the large variation in each year.

Although the hybrid models with a maximum of 7 and 10 input nodes have the smallest RMSE for the training set, these two models frequently have the largest RMSE for the testing set. This likely suggests that the hybrid model, especially with a large number of input nodes, can suffer from overfitting. These models try to capture the variation and seem to look for a pattern in the variation, but any pattern that may exist in the variation of the training set does not necessarily hold true in the testing set. The hybrid model often forecasts a large number of mass shootings (e.g., 7 or 8) in one year followed by a small number (e.g., 3 or 4) in the following year. The experiments reveal that the RMSEs for the testing set for the change-point model, the ARIMA model, and the hybrid model with a maximum of 3 input nodes increase as fewer data points are included in the training set, or equivalently as more data points are included in the testing set. The RMSEs for the testing set for the other hybrid models do not show a trend but vary a lot. The hybrid models with a maximum of 5 and 7 input nodes have the smallest test RMSE of all the models when the training set has the largest number of data points. This result may not be generalizable, however, especially because the hybrid model with a maximum of 10 input nodes has the largest test RMSE for that same training set.

This article is unique in that it compares different forecasting models to predict the number of mass shootings in the future. Comparing different forecasting models sheds insight into the advantages and disadvantages of each model. The hybrid ARIMA-ANN model can be tuned to follow variation in the data, but the pattern of the variation may not continue into the future. The mean of the change-point model and the ARIMA model exhibit much more less annual variation and are not influenced as much by the inclusion of a single data point.

## 5 Appendix

Tables 2 and 3 show the annual count data and the time data of mass shootings generated from the Violence Project database.

**Table 2:** Year count data of mass shootings from the Violence Project.

Year	Number of mass shootings	Year	Number of mass shootings
1966	2	1993	6
1967	1	1994	2
1968	1	1995	3
1969	1	1996	2
1970	1	1997	4
1971	0	1998	4
1972	2	1999	8
1973	1	2000	3
1974	0	2001	4
1975	1	2002	1
1976	1	2003	4
1977	3	2004	3
1978	1	2005	4
1979	0	2006	4
1980	3	2007	4
1981	2	2008	5
1982	3	2009	5
1983	3	2010	5
1984	4	2011	4
1985	1	2012	6
1986	1	2013	5
1987	1	2014	3
1988	3	2015	6
1989	2	2016	5
1990	1	2017	7
1991	5	2018	8
1992	4	2019	8

**Table 3:** Occurring time data of mass shootings from the Violence Project.

*i*th event	*t*_*i*_(days)	*i*th event	*t*_*i*_(days)	*i*th event	*t*_*i*_(days)	*i*th event	*t*_*i*_(days)	*i*th event	*t*_*i*_(days)
1	0	36	8081	71	11944	106	15163	141	17988
2	101	37	8201	72	11944	107	15197	142	18006
3	447	38	8438	73	11987	108	15294	143	18006
4	595	39	8717	74	12043	109	15361	144	18089
5	979	40	9194	75	12089	110	15573	145	18108
6	1512	41	9200	76	12136	111	15577	146	18201
7	2128	42	9215	77	12194	112	15785	147	18226
8	2150	43	9223	78	12279	113	15789	148	18302
9	2351	44	9228	79	12317	114	15813	149	18410
10	3136	45	9354	80	12555	115	15942	150	18440
11	3631	46	9400	81	12573	116	16005	151	18486
12	3848	47	9564	82	12599	117	16062	152	18559
13	4007	48	9587	83	12747	118	16073	153	18675
14	4040	49	9825	84	12812	119	16100	154	18709
15	4336	50	9860	85	13011	120	16222	155	18718
16	4932	51	9928	86	13349	121	16431	156	18797
17	5071	52	9976	87	13482	122	16460	157	18813
18	5100	53	9981	88	13531	123	16496	158	18825
19	5391	54	9988	89	13588	124	16671	159	18881
20	5550	55	10179	90	13841	125	16729	160	18907
21	5752	56	10370	91	13980	126	16779	161	18947
22	5848	57	10467	92	13997	127	16794	162	19021
23	5859	58	10573	93	14095	128	16846	163	19066
24	6027	59	10723	94	14096	129	16923	164	19076
25	6055	60	10778	95	14105	130	17017	165	19157
26	6275	61	10853	96	14262	131	17055	166	19179
27	6496	62	11333	97	14419	132	17101	167	19285
28	6538	63	11359	98	14474	133	17150	168	19347
29	6557	64	11437	99	14530	134	17200	169	19348
30	6563	65	11452	100	14661	135	17359	170	19375
31	6800	66	11535	101	14796	136	17452	171	19474
32	7319	67	11553	102	14860	137	17603	172	19474
33	7567	68	11553	103	15089	138	17841	173	19555
34	7865	69	11609	104	15093	139	17870		
35	8016	70	11904	105	15156	140	17945		

Tables 4–7 present the architecture selection results of the ANN model with different maximum number of input nodes for the different training sets.

**Table 4:** Architecture selection results of the ANN model for different training sets with maximum three input nodes.

Training set observation period	The nubmer of inputs	The number of hidden nodes
1966-2003	1	3
1966-2004	3	3
1966-2005	2	3
1966-2006	4	2
1966-2007	3	3
1966-2008	2	3
1966-2009	2	3
1966-2010	4	3
1966-2011	3	3
1966-2012	3	3
1966-2013	1	3
1966-2014	1	3

**Table 5:** Architecture selection results of the ANN model for different training sets with maximum five input nodes.

Training set observation period	The nubmer of inputs	The number of hidden nodes
1966-2003	4	4
1966-2004	4	5
1966-2005	4	5
1966-2006	4	5
1966-2007	4	5
1966-2008	4	5
1966-2009	3	4
1966-2010	4	5
1966-2011	4	5
1966-2012	4	4
1966-2013	3	3
1966-2014	4	4

**Table 6:** Architecture selection results of the ANN model for different training sets with maximum seven input nodes.

Training set observation period	The number of inputs	The number of hidden nodes
1966-2003	6	7
1966-2004	4	7
1966-2005	6	7
1966-2006	9	7
1966-2007	8	7
1966-2008	6	7
1966-2009	8	7
1966-2010	5	7
1966-2011	5	7
1966-2012	5	6
1966-2013	4	7
1966-2014	9	7

**Table 7:** Architecture selection results of the ANN model for different training sets with maximum ten input nodes.

Training set observation period	The number of inputs	The number of hidden nodes
1966-2003	7	10
1966-2004	7	9
1966-2005	6	9
1966-2006	9	9
1966-2007	7	9
1966-2008	4	9
1966-2009	4	10
1966-2010	5	10
1966-2011	7	10
1966-2012	8	10
1966-2013	5	10
1966-2014	8	9
